# Evaluation of Seasonal and Climatic Variations Associated With Femoral Fracture Incidence Among the Elderly Population in Brazil

**DOI:** 10.7759/cureus.66954

**Published:** 2024-08-15

**Authors:** Ana Beatriz I Horita, Rafael Vargas Silva, Tulio M Ramos dos Santos, Caio R Almeida Lima

**Affiliations:** 1 Orthopedics and Traumatology, Pontifícia Universidade Católica de São Paulo, Sorocaba, BRA

**Keywords:** elderly in brazil, fragility fracture, winter, seasonality, femur fracture

## Abstract

Introduction: Femur fractures represent a significant public health concern for individuals over 60 years of age. In Brazil, the incidence of these fractures has shown a rising trend in line with population aging. Besides intrinsic risk factors like osteoporosis, seasonal and climatic variables have been suggested to significantly influence the occurrence of these fractures.

Methods: Epidemiological data were sourced from the Hospital Information System of the Unified Health System (SIH-SUS) using the TABNET tool provided by the Department of Informatics of the Unified Health System (DATASUS). Information on hospital admissions for femur fractures in individuals over 60 years, categorized by diagnosis, region, and month, was collected from 2008 to 2023. Climatic data were obtained from the Brazilian National Institute of Meteorology (INMET) for the period from 1991 to 2020. Monthly average temperatures were compared with epidemiological data and analyzed to uncover correlations using ANOVA and Tukey's honestly significant difference (HSD) test.

Results: During Brazil's winter months (June to August: 22.8 to 23.5º Celsius), average temperatures are at their lowest. This period also coincides with the peak incidence of femur fractures among the elderly. ANOVA revealed significant differences in fracture rates across various temperature ranges. Subsequent analysis using Tukey’s HSD test identified significant differences between temperature ranges of 22-23°C and 25-26°C, as well as 23-24°C and 25-26°C. These results indicate that lower temperatures are associated with a higher incidence of femur fractures among the elderly.

Conclusion: The analysis reveals a seasonal pattern in fracture incidence among older adults, with a notable increase during the colder months. To mitigate this risk, it is recommended to implement strategies such as heightened surveillance during colder months, targeted fall prevention measures, and effective osteoporosis management. These interventions aim to reduce the incidence of fractures in this vulnerable population.

## Introduction

Femur fractures represent a serious public health problem, especially in individuals over 60 years old, due to the associated complications and increased mortality. Generally, the incidence of these fractures is higher in females [1], and in Brazil, the overall incidence has been increasing, in line with the aging population. In addition to risk factors such as osteoporosis and low-energy trauma, studies suggest that climatic variables may play a significant role in the occurrence of these fractures [2].

In Brazil, a country with great climatic diversity, it is crucial to understand how different climatic conditions affect the incidence of femur fractures in the elderly. The five regions of Brazil exhibit significant variations in terms of temperature, humidity, and precipitation, offering an opportunity to study the influence of climate on the bone health of the elderly. For instance, the Southern region of Brazil experiences greater temperature amplitudes compared to the Northeastern region, which has a warmer and drier climate throughout the year [3].

Although climate is not a modifiable factor, it is important to identify and study the seasonality of these fractures in Brazil, as no Brazilian data has been published so far. Thus, through epidemiological data from the Hospital Information System of the Unified Health System (SIH-SUS), available from DATASUS/TABNET, and meteorological data provided by the National Institute of Meteorology of Brazil, this study aims to outline the climatological profile of femur fractures in the elderly in Brazil. 

## Materials and methods

This study utilized a quantitative and qualitative approach to investigate the correlation between the incidence of femoral fractures in individuals over 60 years old and climate variations in Brazil.

Epidemiological data were obtained from the Hospital Information System of the Unified Health System (SIH-SUS), using the TABNET tool provided by the Department of Informatics of the Unified Health System (DATASUS). Initially, the official SIH-SUS website was accessed and the TABNET tool was used to access the "Epidemiological and Comorbidities" section [4]. Then, the "SUS Hospital Morbidity (SIH/SUS)" option was selected by hospitalization location, in order to separate the data by each region of Brazil: Northeast, North, Central-West, South, and Southeast.

Thus, a table was obtained with the total number of hospitalizations for femoral fractures in the elderly, divided by each region of the country, by each month of the year. Data from the period of January 2008 to December 2023 were recorded.

Meteorological data were provided by the National Institute of Meteorology (INMET), through the table "Climatological Norms of Brazil: from 1991 to 2020", covering the study period. The period 1991-2020 was selected on the official INMET website [5]. Subsequently, in the "Temperature" section, the item "Monthly and Annual Average Compensated Temperature (°C)" was chosen, and the table with values from each meteorological station in the country, separated by month, was downloaded. Next, the values were summed for each month of the year, resulting in the average monthly temperature for each month in the country.

Using this data, a column chart was created to represent month-to-month values, and these same values were also plotted on a comparative graph for analysis. The collected data were organized into a single database to allow integrated analysis to investigate the correlation between the epidemiological and meteorological variables. The following steps were followed: The data were structured in a suitable format for analysis, with the variables of interest (number of fractures and average monthly temperature) organized by year and month. An initial descriptive analysis was conducted to summarize the main characteristics of the data, including means, standard deviations, and distributions of the variables over the study period. Time series graphs were generated to visualize trends in temperature and the incidence of fractures over the years and months.

For data analysis, TABNET/DATASUS software was used for the extraction and manipulation of epidemiological data; additionally, Microsoft Excel (Microsoft Corp., Redmond, WA) was used for standard deviation, as well as for the preliminary organization of data and initial calculations.

Sequentially, analysis of variance (ANOVA) was used to explore the association between five distinct temperature groups and the incidence of femur fractures. The ANOVA outcomes uncovered a notable disparity in femur fracture tallies across different temperature ranges. Subsequently, in-depth investigations into specific temperature bins were conducted using Tukey's honestly significant difference (HSD) test. This post hoc assessment involved pairwise comparisons among temperature groups to discern significant discrepancies in mean fracture counts. The statistical analysis was implemented with Python, leveraging libraries such as Pandas, Numpy, and Statmodels.

## Results

The following table presents data on the number of hospitalizations for femoral fractures among the Brazilian elderly, categorized by each month from January 2008 to December 2023. Additionally, the climatological norms for the same months are provided (Table 1).

**Table 1:** Raw Data of the Monthly Median Temperatures and Femur Fracture Incidence.

Month	Median Temperatures (º C)	Femur Fractures
January	25.9	64789
February	25.8	58449
March	25.8	64628
April	25.5	65014
May	24	74065
June	22.8	74836
July	22.2	80476
August	23.5	78313
September	24.8	70546
October	26	70672
November	25.7	66380
December	25.7	67716

The table demonstrates the number of femur fractures in patients older than 60 years as collected at DATA-SUS and the median temperature in each month according to INMET.

**Figure 1:**
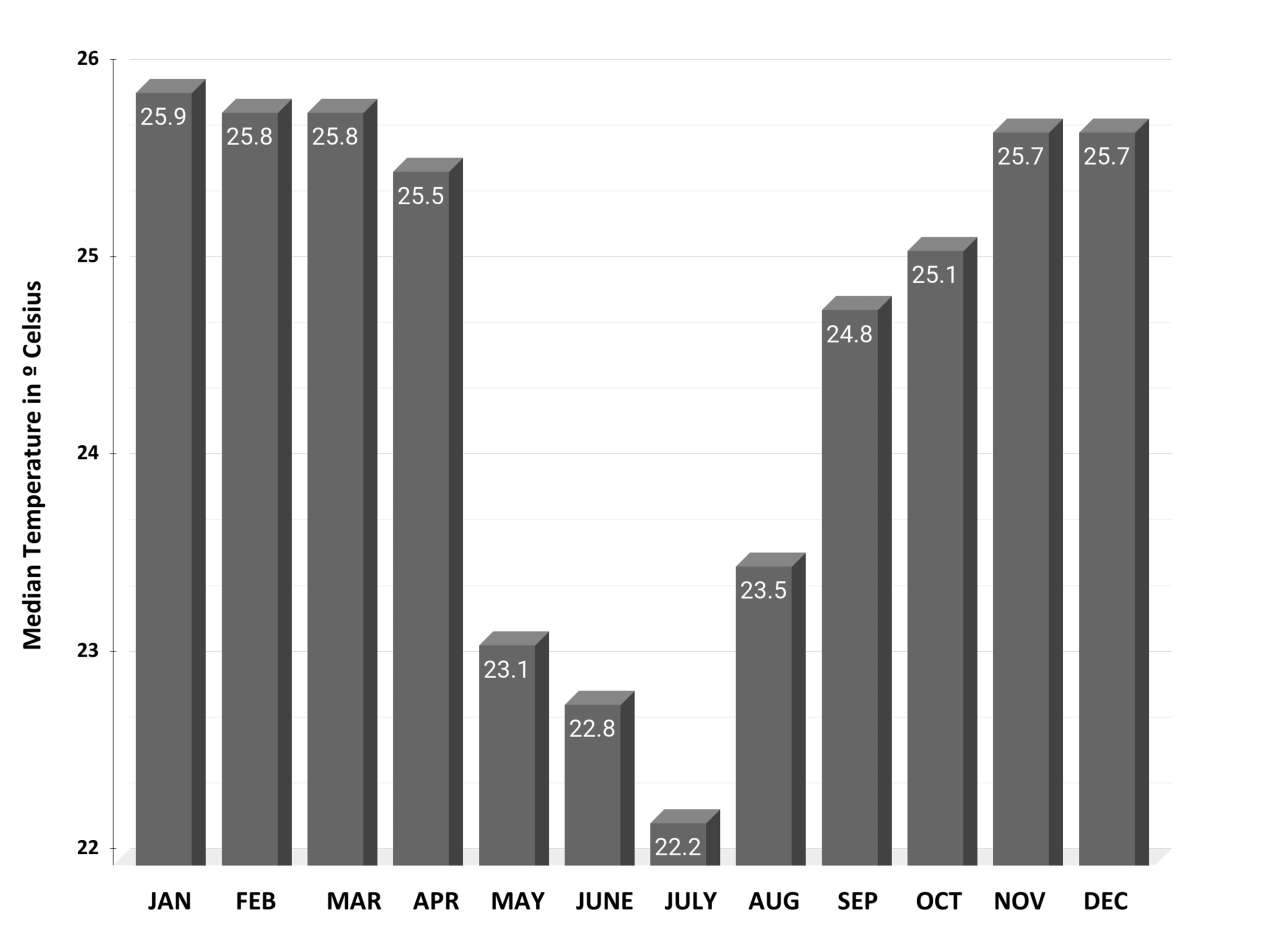
Monthly Average Temperature Distribution in Brazil. This graph illustrates the climatological data, highlighting the variation in average temperatures across different months from January to December.

The climatographic data of Brazil (Figure 1) showcases the monthly average temperatures throughout a calendar year, highlighting the distinct climatic variations across different months.

The analysis reveals a significant seasonal fluctuation in temperatures across the year. Key observations from the data include that January registers the highest average temperatures, peaking at approximately 26°C. This trend continues through February, maintaining elevated temperatures. February, notable for its high temperatures, averages around 25°C to 26°C and is characterized by high humidity levels and increased rainfall in many regions.

A gradual decrease in temperatures is observed as Brazil transitions from summer to autumn. March temperatures average around 25°C, descending from approximately 23°C to 22°C by May. The reduction in temperatures corresponds with a relative decrease in humidity and a marked reduction in precipitation in several Brazilian regions, signifying the shift towards the drier season.

Winter months in Brazil show the most significant drop in temperatures. June marks the beginning of winter with temperatures averaging around 22.8°C in some of the climatic stations evaluated. July, the peak of winter, displays the lowest average temperatures of the year, falling to approximately 22.2°C in the most cold stations evaluated. August sees a slight rise in temperatures as Brazil transitions into spring, with averages going up again. The pronounced cooling during these months is more evident in the southern regions of Brazil, such as Rio Grande do Sul, Santa Catarina, and Paraná, where temperatures can drop to freezing levels, occasionally resulting in frost and snowfall in elevated areas.

A noticeable warming trend begins in the spring in September, with temperatures rising to around 24°C even in the most cold stations, marking the end of winter. October continues this warming trend, with average temperatures climbing back up to approximately 25°C, indicating the onset of the austral spring. By November, temperatures rise further to about 26°C, nearing the high summer averages once again. These months are characterized by increasing humidity and the return of higher precipitation levels, especially in central and southeastern Brazil.

**Figure 2:**
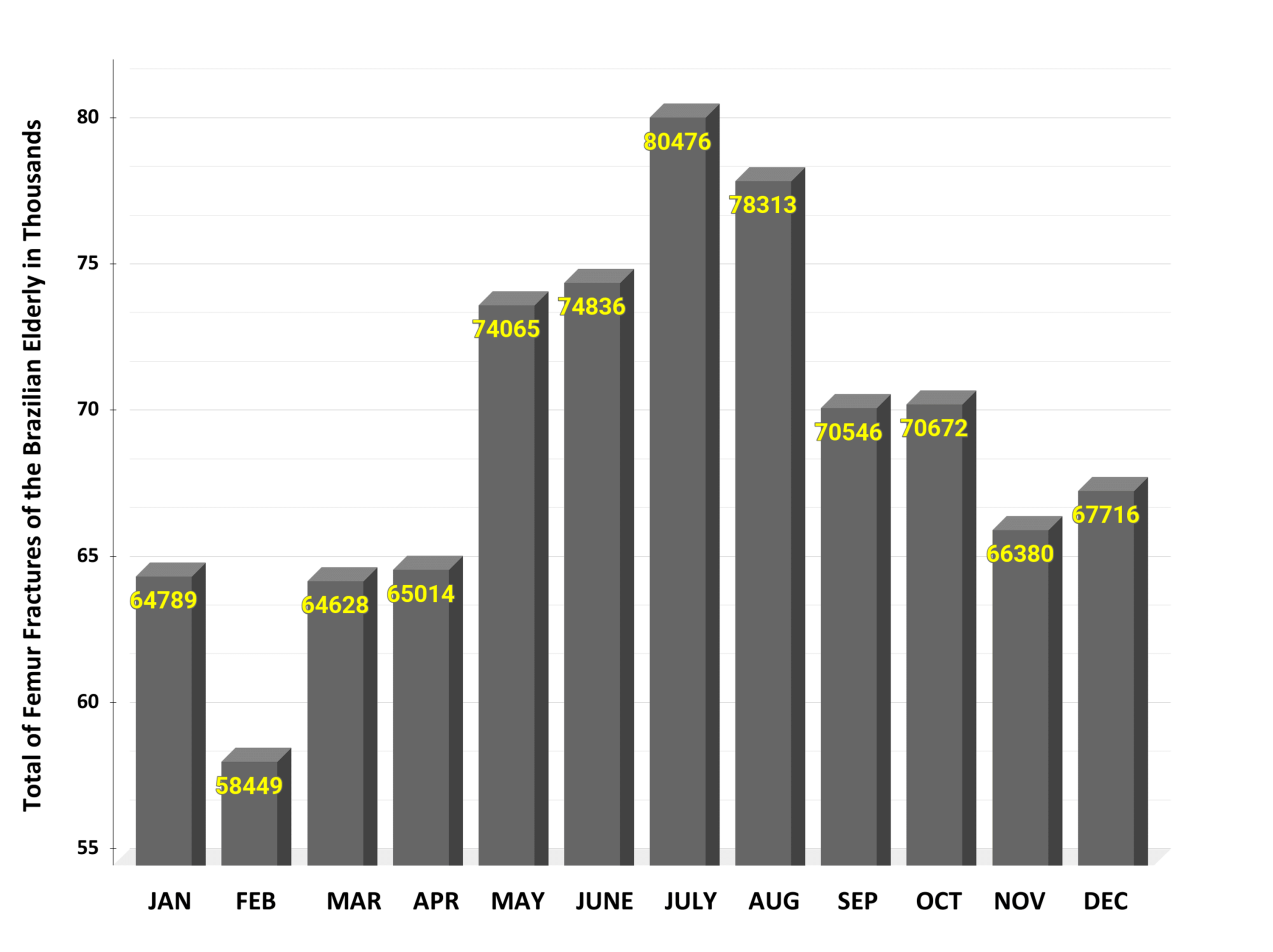
Monthly Incidence of Femur Fractures in Individuals Aged 60 and Over (2008-2023). This graph displays the number of femur fractures reported each month over a 15-year period, highlighting seasonal trends in fracture occurrences.

The graph in Figure 2 presents the monthly distribution of femur fractures in individuals aged 60 and above over a 15-year period.

In January, the number of fractures is approximately 64,000 every year. February sees a slight decrease with around 58,000 fractures. March presents a notable increase with around 64,000 fractures. Further increase in April, reaching close to 65,000. May sees an uptick to nearly 74,000 fractures. The increase in May could be related to changing weather patterns causing unexpected hazardous conditions.

A significant rise occurs in June, approaching nearly 74,000 fractures. The highest number of fractures is observed in July, reaching approximately 80,000. August shows a decline, with the number of fractures dropping to around 78,000. September and October continue the decline, with fractures numbering around 70,000. November, approximately 66,000 fractures.

December shows a slight increase, with fractures reaching approximately 67,000. This increase might be due to holiday-related activities and gatherings, which could lead to a higher incidence of falls among the elderly.

**Figure 3:**
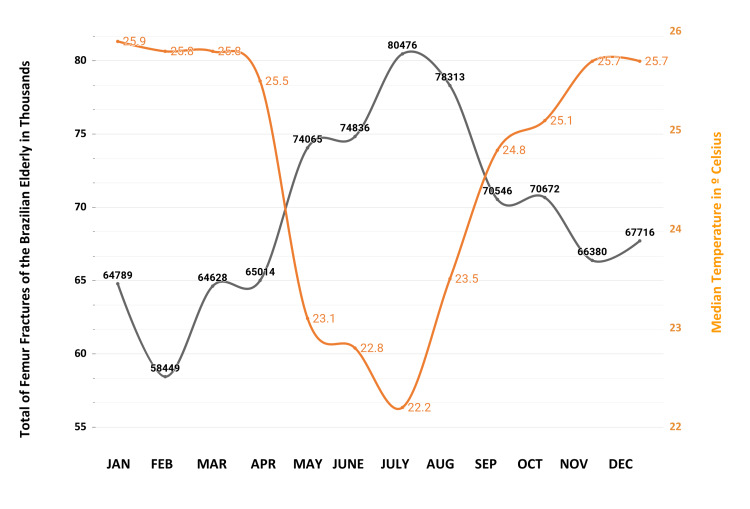
Overlay of Femur Fracture Incidence and Average Monthly Temperature in Brazil. This graph compares the monthly patterns in femur fracture cases (indicated by the gray line) with the corresponding average temperatures (indicated by the orange line), revealing potential correlations between climate and fracture frequencies.

The graph in Figure 3 illustrates the monthly distribution of femur fractures in individuals aged 60 and above from 2008 to 2023, alongside average monthly temperatures in Brazil.

The winter months in Brazil (June to August) have the lowest temperatures of the year. June begins the winter with averages of 22.8°C, July shows the lowest average of the year around 22.2°C, and August begins to show a slight increase, while there is a significant increase in the number of fractures.

Following, we describe the analysis of variance (ANOVA) employed to investigate the correlation between different temperature categories and the incidence of femur fractures. The raw data, categorized into groups, is presented in Table 2 below.

**Table 2:** Months by Temperature Categories and Femur Fracture Incidence. The table demonstrates the number of femur fractures in patients older than 60 years in each month and the breakdown of each month between temperature groups.

Month	Femur Fractures	Temperature Category (º C)
January	64789	25-26
February	58449	25-26
March	64628	25-26
April	65014	25-26
May	74065	24-25
June	74836	22-23
July	80476	22-23
August	78313	23-24
September	70546	24-25
October	70672	26-27
November	66380	25-26
Dezember	67716	25-26

The outcomes of the ANOVA test are presented in Table 3. The ANOVA results illustrated a significant discrepancy in the occurrences of femur fractures across different temperature ranges and revealed a sum of squares of 377,769,200 for Temperature and 72,952,140 for Residual. The Degrees of Freedom were 4 for Temperature and 7 for Residual. The computed F-statistic was 9.062052, with a corresponding p-value of 0.00671.

**Table 3:** ANOVA outcome.

Source	Sum of Squares	Degrees of Freedom	F-Statistic	Corresponding P-Value
Temperature (º C)	3.78E+08	4	9.062052	0.00671
Residual	7.30E+07	7		

In the analysis, the p-value of 0.00671 falling below the standard threshold of 0.05 signifies robust statistical evidence to refute the null hypothesis, highlighting a significant difference in the means of femur fractures across different temperature ranges. Furthermore, the high F-statistic of 9.062052, coupled with the low p-value, indicates substantial variation between temperature categories compared to within-range variations, highlighting the significant influence of temperature classifications on femur fracture counts.

The subsequent analysis delved deeper into specific temperature ranges using Tukey’s HSD test. This comprehensive post hoc assessment involved performing pairwise comparisons between temperature groups to identify substantial variations in mean fracture counts. Below, we present the outcome of the Tukey’s HSD test in the Table 4:

**Table 4:** Tukey’s HSD test outcome.

Comparison Temperature Group 1 (º C)	Comparison Temperature Group 2 (º C)	Mean Difference	Adjusted P-Value	Lower Limit	Upper Limit	Reject the Null Hypothesis
22-23	23-24	657	0.9998	-13489.78	14803.78	FALSE
22-23	24-25	-5350.5	0.5107	-16901.3	6200.3	FALSE
22-23	25-26	-13160	0.0096	-22591.19	-3728.81	TRUE
22-23	26-27	-6984	0.457	-21130.78	7162.78	FALSE
23-24	24-25	-6007.5	0.5824	-20154.28	8139.28	FALSE
23-24	25-26	-13817	0.0312	-26293.29	-1340.71	TRUE
23-24	26-27	-7641	0.5025	-23976.29	8694.29	FALSE
24-25	25-26	-7809.5	0.1084	-17240.69	1621.69	FALSE
24-25	26-27	-1633.5	0.9925	-15780.28	12513.28	FALSE
25-26	26-27	6176	0.4547	-6300.29	18652.29	FALSE

Regarding Tukey’s HSD data, distinctions were observed when comparing 22-23 °C and 25-26°C, showing a mean difference (MD) of -13.160, with an adjusted p-value of 0.0096 and a 95% CI -22,591.19, -3,728.81. Similarly, when examining 23-24°C versus 25-26°C, a significant difference was found, with an MD of -13.817; an adjusted p-value of 0.0312, and a 95% CI of -26,293.29, -1,340.71. Furthermore, no statistically significant differences were observed in the comparisons between the remaining groups.

Regarding the incidence of femur fractures among the elderly in Brazil, the final pooled analysis of the data reveals that the incidence is statistically significantly higher during the lower temperature months compared to the higher temperature months.

## Discussion

The results demonstrated that in Brazil there is a tendency for a higher number of hospitalizations of elderly individuals due to femur fractures during the colder months, which aligns with the existing literature from other countries such as the United States of America, Canada, the United Kingdom, Sweden, Australia, Spain, Italy, Hong Kong, and South Korea [2,6-18]. Similar to Mazzucchelli et al., who conducted a study about the effects of seasonality on osteoporotic fractures in the elderly at a hospital in Madrid, Spain [19], it was observed that the increase in hospitalizations began in the autumn months. This Madrid study states that the highest incidence of hospitalizations occurred during the European autumn, whereas in the present study conducted in Brazil, the highest numbers of hospitalizations were recorded in the winter. This difference may be attributed to the fact that Mazzucchelli et al. included osteoporotic fractures such as distal radius and proximal humerus in their study, and the hypothesis that in Spain, the elderly continue to leave their homes for physical and daily activities during the autumn, which, despite low temperatures, are not as extreme as in the winter, when the elderly rarely leave their homes, reducing the number of falls outside their homes [19].

Moreover, in Brazil, we do not observe winter months with extremely low temperatures. It is evident that there are significant climatic variations within Brazilian territory, with the southern region experiencing the lowest temperatures and the greatest thermal amplitudes throughout the year, and the northern region experiencing a higher temperature year-round and the smallest thermal amplitude.

In the literature, most published data were collected from and describe countries at higher latitudes and north of the equator compared to Brazil. Therefore, these countries experience harsh winters with frequent snow and frost. The only region in Brazil that can be compared is the South, as it is located at a similar latitude to other studied countries. Ortiz et al. [20] conducted a systematic review comparing 20 studies related to this topic, confirming agreement with our study's results. They established a causal relationship between the increased incidence of proximal femur fractures during the colder months and the increased number of falls during these periods. However, it was not possible to define a correlation with aspects of bone metabolism, such as variations in sun exposure, vitamin D levels, and parathyroid hormone throughout the year.

In a study conducted in Sydney, Australia [17], Chiu et al. found an inverse relationship between daily average temperature and the risk of hip fractures, establishing that in warm climates, physical activity and sun exposure are protective factors.

It is important to note that the population over 60 years old suffers much more acutely and multifaceted effects of climatic variations compared to younger individuals. A brief period during which the elderly are unable to perform their usual physical or strengthening activities can lead to a rapid loss of muscle mass [20], impairing balance, reducing performance status and autonomy, and exponentially increasing the risk of falls both inside and outside their homes [21].

In addition to the intrinsic mechanism of falls, it is essential to mention that bone metabolic factors must be evaluated as important variables [22]. For example, lower sun exposure during the winter results in lower levels of active vitamin D, which can demineralize bones and increase the risk of fractures.

The nutritional status of the elderly directly affects the risk of osteoporosis, with persistent problems in regions such as the North and Northeast of Brazil. Poor calcium and macronutrient intake due to inadequate diet makes individuals more susceptible to deficiency diseases, with osteoporosis being one of them. Proximal femur fractures in the elderly are considered typical osteoporotic fractures [23], presenting devastating consequences as they are associated with high mortality and morbidity. In resource-poor regions, such as the interior of the North and Northeast of Brazil, limited access to care further exacerbates the situation. The treatment of hip fractures in the elderly involves surgical intervention, with synthesis material or arthroplasty and skilled surgeons, early postoperative physiotherapy, nutritional supplementation of calcium and vitamin D, and medicinal therapy including antibiotics and prophylaxis against thromboembolic events, indicating a multidisciplinary approach. A deficient healthcare infrastructure is incapable of adequately addressing these fractures, increasing the risks of unfavorable outcomes and failing to treat one of the country's most severe public health issues, which is osteoporosis.

Given this, there are inherent limitations to this study, including possible confounding factors that were not controlled. Additionally, the quality and accuracy of meteorological and epidemiological data depend on the original data source, which may exhibit regional variations in data collection and reporting.

Recommendations

As this study has established a seasonal pattern regarding femur fracture incidence in the elderly, this should be addressed with targeted strategies such as increased surveillance and support during the colder months, increased awareness of fall prevention strategies, provision of non-slip footwear, and ensuring adequate lighting in areas where older people move around. In addition, effective programs to manage and treat osteoporosis are essential.

## Conclusions

The analysis clearly indicates a seasonal pattern in fracture incidence among older adults, with a significant increase in incidence during colder months. Given this, this correlation should be addressed with targeted strategies such as heightened surveillance during high-risk periods, year-round health initiatives, and improvements to older adults’ community and home environments. With a multifaceted approach, there is an opportunity to substantially reduce the incidence and impact of fractures in this vulnerable demographic.
